# Operative Outcomes for Cervical Myelopathy and Radiculopathy

**DOI:** 10.1155/2012/919153

**Published:** 2011-10-20

**Authors:** J. G. Galbraith, J. S. Butler, A. M. Dolan, J. M. O'Byrne

**Affiliations:** ^1^Department of Trauma and Orthopaedic Surgery, Cork University Hospital, Wilton, Cork, Ireland; ^2^Department of Trauma and Orthopaedic Surgery, Cappagh National Orthopaedic Hospital, Finglas, Dublin 11, Ireland

## Abstract

Cervical spondylotic myelopathy and radiculopathy are common disorders which can lead to significant clinical morbidity. Conservative management, such as physical therapy, cervical immobilisation, or anti-inflammatory medications, is the preferred and often only required intervention. Surgical intervention is reserved for those patients who have intractable pain or progressive neurological symptoms. The goals of surgical treatment are decompression of the spinal cord and nerve roots and deformity prevention by maintaining or supplementing spinal stability and alleviating pain. Numerous surgical techniques exist to alleviate symptoms, which are achieved through anterior, posterior, or circumferential approaches. Under most circumstances, one approach will produce optimal results. It is important that the surgical plan is tailored to address each individual's unique clinical circumstance. The objective of this paper is to analyse the major surgical treatment options for cervical myelopathy and radiculopathy focusing on outcomes and complications.

## 1. Introduction

Spondylosis is the most common cause of neural dysfunction in the cervical spine and is becoming more prevalent as the average life-expectancy increases [1]. The degenerative changes associated with ageing include disc herniation, osteophyte formation, hypertrophy of osteoarthritic facet joints, and hypertrophy of ligaments. This condition is often asymptomatic, but in 10% to 15% of cases it compresses the cervical spinal cord and roots to present symptomatically as myelopathy or radiculopathy [2, 3]. Conservative management, such as physical therapy, cervical immobilisation, or anti-inflammatory medications are the preferred and often only required intervention [4]. Surgical intervention is reserved for those patients who have intractable pain or progressive neurological symptoms. Herniated cervical discs and spondylosis causing radiculopathy may be treated from an anterior or posterior approach. Likewise, decompression of the spinal cord can be achieved from either approach. The goals of surgical treatment are decompression of the spinal cord and nerve roots and deformity prevention by maintaining or supplementing spinal stability and alleviating pain. 

Many surgical techniques have been described to decompress the spinal cord and roots which can employ an anterior, posterior, or circumferential approach. Under most circumstances, one approach will produce optimal results [5–10]. Designing the most effective surgical plan is dependent on numerous factors, including the location of the compressive pathology, stability of the spinal column, extent of the disease, medical comorbidity, and the surgeon's experience and comfort level with specific procedures. The objective of this paper is to analyse the major surgical treatment options for cervical myelopathy and radiculopathy focusing on outcomes and complications.

## 2. Treatment Outcomes for Cervical Spondylotic Myelopathy

### 2.1. Posterior Surgical Techniques

A posterior approach is best utilised when pathology is present dorsally in the spinal canal. It avoids extensive dissection of vital neck structures and graft-related complications encountered with anterior approaches. Major approach-related complications include postoperative pain from injury to paraspinal muscles, epidural haematoma, and neurological injury. It is contraindicated in a kyphotic deformity, and there is limited potential for open deformity reduction with the more common posterior fixation techniques [6, 11–14]. 

#### 2.1.1. Laminectomy

Laminectomy has been proven to be a safe and effective technique for multilevel decompression for cervical spondylotic myelopathy (CSM) [12, 15]. Laminectomy without fusion has demonstrated comparable immediate postoperative results to laminoplasty and anterior procedures [6]. There is a body of evidence, however, demonstrating late deterioration, with rates as high as 40% [6, 10, 16]. 

Miyazaki and Kirita described 155 patients who underwent multilevel laminectomy [17]. They reported an improvement in JOA (Japanese Orthopaedic Association) outcome scale for 82% of patients at a mean followup of 1 year. Eleven percent reported worsening symptoms and 7% remained the same. Ebersold et al. reported outcomes in 51 patients who underwent laminectomy for myelopathy [6]. At 6 months, 69% showed improvement on the Nurick scale but only 37% sustained the improvement at long-term followup (range 3 to 9.5 years). 

Neurological injury is a rare but serious complication of this procedure. The incidence of spinal cord injury is from 0% to 3%, whereas injury to an individual nerve root can be as high as 15% [18, 19]. Nerve root injury occurs due to direct manipulation or dorsal migration of the spinal cord after decompression [19]. Epidural haematoma occurs in 0.08 to 1.3% of cases [13]. Postlaminectomy kyphosis and segmental instability are well-documented complications which have led to the limited indication of laminectomy alone as a surgical option for CSM. This postlaminectomy spinal instability is reported to occur in 14–47% of patients [9, 10, 20]. This has not been shown to be associated with the observed delayed neurological deterioration [9, 10, 20, 21]. Kato et al. described the JOA outcome in 44 patients who underwent laminectomy. There was 44.2% recovery rate at 1 year that decreased to 43% at 5 years and 33% at 10 years. There was a 47% rate of postoperative kyphosis and a 23% incidence of late deterioration (mean 9.5 years). The development of kyphosis did not appear to correlate with neurological deterioration in these cases [10].

Laminectomy can be augmented to include posterior instrumentation to address instability which leads to lower rates of kyphosis and segmental instability. Despite this increased stability, addition of instrumentation can lead to complications such as hardware failure with loss of alignment and neurological damage from misplaced lateral mass screws. Heller et al. from their series reported a 1% risk of nerve root injury per screw placed [22]. Adjacent segment degeneration can also occur due to alterations to cervical biomechanics and force distribution following a fusion procedure [10]. Houten et al. evaluated 38 patients who underwent laminectomy and lateral mass plating for CSM [8]. Significant improvement in neurological function occurred in 97% of patients. The modified JOA score improved from 12.9 to 15.6 at a mean followup of 30.2 months (minimum 6-months). Radiographic alignment by the cervical index was maintained postoperatively. Complications included a C-5 nerve root palsy, a radiculopathy from a misplaced screw, and one wound infection. Huang et al. reviewed 31 patients who underwent laminectomy and lateral mass plating for CSM or OPLL [23]. Twenty-two (71%) of 31 patients had improvement in Nurick score of ≥1 point at a minimum of 6 months of followup (mean 15 months). Perez-Lopez et al. compared 19 patients that underwent laminectomy to 17 that underwent laminectomy and fusion [24]. They found similar improvement in Nurick scores but, there was an increase in postoperative kyphosis with in the laminectomy alone cohort (24%) compared to the laminectomy and fusion group (7%).

#### 2.1.2. Laminoplasty

Laminoplasty was developed to allow cord decompression while preserving motion with less substantial alteration to the natural biomechanics of the cervical spine. Multiple studies have demonstrated its effectiveness using the JOA outcome scale, with approximately 55–65% achieving recovery [25–30]. 

 In the short term, Kihara et al. reported on 151 patients with CSM who underwent laminoplasty [28]. The mean JOA scale score increased from 8.1 to 15.2 at 1-year followup. Similarly, Suda et al. reported on 154 patients with CSM who underwent French-window laminoplasty [29]. The JOA scale score improved from 9.9 to 14.0 (60% improvement) at a mean followup of 5 years. In the longer term, Seichi et al. reviewed 60 patients (35 with OPLL and 25 with CSM) who underwent French-window laminoplasty [27]. In the OPLL group, the JOA scale score increased from 8.6 to 12.1; similar increases were seen in the patients with CSM (improvement from 8.3 to 12.0) at followup of 10 years. Late clinical worsening was observed in 11 patients (7 with OPLL and 4 with CSM).

Several variations of laminoplasty have been described in order to minimise complications [11]. Okada et al. recently carried out a prospective randomised clinical study comparing open-door laminoplasty to French-door laminoplasty in 40 patients [26]. JOA scores and recovery rates were similar at long-term followup (mean 26.9 months). However, French-door laminoplasty had less complications, increased cervical lordotic angle, and significantly less axial neck pain postoperatively suggesting that this may be a better procedure for CSM.

Decreased range of movement and axial neck pain are frequently reported complications. Ratliff and Cooper performed a meta-analysis evaluating the outcomes after laminoplasty in 71 retrospective reports [30]. They determined the overall incidence of postoperative axial neck pain ranged from 6% to 60% without apparent dependence on the specific variation of laminoplasty. Cervical range of movement decreased substantially after laminoplasty (mean decrease 50%, range 17–80%). Wada et al. performed a study comparing subtotal corpectomy and laminoplasty Axial pain was observed in 15% of the corpectomy group and in 40% of the laminoplasty group [25]. The aetiology of neck pain remains unclear, but has been postulated to occur secondary to neck muscle disruption, particularly detachment of muscle insertions to the C2 and C7 spinous processes [31, 32]. Other authors have reported that preservation of subaxial deep extensor muscles, including the semispinalis cervicis groups, reduces these adverse effects after cervical laminoplasty [33]. However, a recent study by Sakaura et al. suggests that preservation of subaxial deep extensor muscles plays no significant role in reducing axial neck pain supporting the idea that axial pain after laminoplasty largely results from detachment of muscles attached to the C2 and C7 spinous processes [34].

### 2.2. Anterior Surgical Techniques

This approach allows direct decompression of ventral pathology but can also be used to restore lordosis to a kyphotic spine. The anterior approach comes in proximity with many vital structures in the neck. Complications resulting from this approach include, dysphagia, recurrent laryngeal nerve damage, dural tears, and rarely tracheal or oesophageal perforation (less than 0.25%) [35, 36].

Postoperative dysphagia has been reported to have an overall average incidence of 12.3% [37, 38]. This dysphagia is usually transient with residual symptoms decreasing to 4.8% after 6 months [39].

#### 2.2.1. Anterior Cervical Discectomy

Anterior Cervical Discectomy with or without fusion is effective for ventral pathology that is confined to the cervical interspaces such as osteophyte or disc complexes. Most of the recent literature has focused on outcomes for Anterior Cervical Discectomy with Fusion (ACDF). Short- and long-term clinical success in the range of 67% to 100% has been extensively reported in the literature [6, 40–47]. Despite the increasing popularity of ACDF, it has not been proven to produce better clinical outcomes than anterior cervical discectomy without fusion (ACD) [48, 49].

Ebersold et al. reported Nurick scale outcomes in 33 patients with ACDF at 1 or 2 levels [6]. Six-month outcomes showed improvements of 73% and long-term improvement of 55% (range 3 to 9.5 years). Yue et al. reviewed 71 patients, after an average of 7.2 years, who had anterior cervical discectomy and fusion with allograft and plating [43]. Patients reported improvement in axial neck pain (95.5%), radicular arm pain (95.4%), upper extremity weakness (82.7%), upper extremity numbness (85.1%), and gait problems (100%). Fusion occurred in 92.6% of the disc spaces operated on and no graft extrusion or migration occurred. Clinical improvements were not related to the occurrence of union.

Autograft from either the iliac crest or fibula is traditionally gold standard for fusion [46]. Donor site morbidity, which occurs in 0.6% to 36% of cases, is a complication of its use [50–52]. This can be avoided with the use of allograft. However, their use comes with potential problems, including risk of infections and graft rejection, higher rates of collapse and nonunion, especially in multilevel fusions, and prolonged period required for graft incorporation [46]. Ryken et al. in a comprehensive review concluded that there appears to be equivalency regarding the use of harvested autogenous bone graft, allograft, polyetheretherketone, and titanium cages in anterior fusion [53].

ACDF of 1 to 3 levels has been reported to be effective and safe in decompressing ventral pathology. The rate of fusion following single-level ACDF generally ranges from 80% to 95% [54–57]. However, applying this procedure to greater than 3 levels can often result in complications, including graft extrusion, subsidence, fracture, and pseudoarthrosis [40]. 

Nirala et al. reviewed 69 patients that underwent multilevel ACDF using autograft iliac crest without fixation. Fusion was assessed on dynamic radiographs. The overall fusion rate for multilevel ACDF was 69.6%, The fusion rate was 86.7% for 2 levels, 57.6% for 3 levels, and 50% for 4 levels. The outcome score using Odom's criteria was good or excellent in 81.1%. Graft dislodgements were noted in 1.4% [54]. Fraser and Härtl performed a systematic review comparing ACDF with anterior cervical corpectomy with fusion (ACCF) [58]. They analyzed a combined group of 2682 patients. They found similar fusion rates (>90%) for 2-level disc disease treated with either 2-level ACDF plus fixation or 1-level ACCF plus fixation. For 3-level disc disease, fusion rates for ACDF with plate fixation (82.5% fusion rate) were significantly lower than for ACCF with plate fixation (96.2% fusion rate).

The use of plating remains a controversial issue. In multilevel ACDFs, studies have demonstrated that rigid plate fixation dramatically increases fusion rates [44, 46, 47]. The aim is to promote solid bone fusion, maintain cervical alignment, decrease need for external orthosis, and prevent graft subsidence, extrusion, or collapse. However, the efficacy of rigid plate fixation on interbody fusion in one-level ACDF is not as clear. Some reports suggest that it can decrease fusion rates due to stress shielding and poor graft settling [42, 45]. Some studies advocate better fusion rates with dynamic plating [59]. Plating can also lead to complications including adjacent level degeneration, soft tissue injury, and implant failure [41].

#### 2.2.2. Anterior Cervical Corpectomy

Anterior cervical corpectomy with fusion (ACCF) is effective in addressing ventral pathology that extends beyond the cervical spine interspaces [58]. ACCF has the potential to allow reduction of kyphotic deformities that exacerbate CSM. Stabilisation after corpectomy is achieved with or without instrumentation using tricortical autogenous iliac bone graft, autogenous, or allogenous fibular graft, and more recently titanium mesh cages (TMC), stackable PEEK (polyetheretherketone), and CFRP (carbon-fibre-reinforced polymer) cages [60, 61]. 

ACCF compares favourably when compared to other decompression techniques in terms of stability and clinical outcomes [58]. Hilibrand et al. reviewed a series of 190 patients with a mean followup of 68 months [62]. There were 131 patients that underwent ACDF using autograft without fixation and 59 patients that underwent ACCF using iliac or fibula strut autograft. The fusion, which was assessed on dynamic radiographs, was higher in patients who underwent ACCF but clinical outcomes using Robinson's criteria were not statistically different between the groups. Wada et al. reviewed 23 patients that underwent ACCF. The average JOA score was 7.9 before surgery, 13.3 at the 1-year follow-up visit, and 13.9 at the 5-year followup visit [25]. Similarly, Yonenobu et al. compared the results of corpectomy, laminectomy, and discectomy and concluded that ACCF provided a better neurologic recovery [63]. They suggested that ACCF should be used for treatment of CSM for up to 3 levels and posterior laminectomy for 4 levels or more.

Single-level corpectomy is generally considered safe and associated with successful outcomes for CSM [64]. However, increasing the number of vertebral bodies resected during a corpectomy is associated with an increase in graft-related complications and pseudoarthrosis [19, 61]. Wang et al. reported 249 consecutive patients underwent one- to five-level anterior cervical corpectomies and autogenous strut grafting [61]. During the follow-up period (mean 4.7 years), 16 patients experienced migration of their grafts. The graft migration rates increased with more levels of fusion. Ikenaga et al. reported a much lower rate of pseudoarthrosis. They reviewed 31 patients that had anterior corpectomy and fibular strut grafting for 4-disc levels or greater for CSM for over 10 years [65]. There were 1 patient that developed pseudoarthrosis and 3 patients who had deterioration of JOA score of 1 point.

Plating has been shown to reduce multilevel corpectomy but also in single level corpectomy [46, 47, 60, 61, 66]. Epstein found that plating can decrease the prevalence of graft-related complications following single level ACCF [66]. He reviewed 48 patients undergoing ACCF, 8 of who had plating. Overall there was a 73% fusion rate but 10.3% of cases required revision surgery for graft-related complications or pseudoarthrosis. Of the 8 patients that had plating there were no graft related complications.

Several articles have evaluated effectiveness of titanium mesh cage for reconstruction following anterior cervical corpectomy. Narotam et al. prospectively evaluated 37 patients over a 4-year period, and noted a stability rate of 100% at 1 year after ACCF with a TMC cage. Cage-related complication rate was low (2.7%). Excellent neurological outcome was documented in 95% of the patients. Similarly, Daubs and Kabir et al. reported similar good short-term results with spinal fusion observed in 100% of patients [60, 67].

## 3. Treatment Outcomes for Cervical Radiculopathy

The objective of operative treatment in cervical radiculopathy is to alleviate pain, decrease sensorimotor deficits, and improve quality of life. This can be achieved by the permanent decompression of the compressed nerve root [5]. Similarly to CSM surgical treatment, treatment options can utilise a posterior or an anterior approach. The procedures achieved through these approaches differ in complexity, duration, and complications [68]. The choice of procedure should depend on the patient's symptoms and the morphology of the pathology.

### 3.1. Posterior Laminoforaminotomy

Posterior laminoforaminotomy is used for decompression of the nerve root in cases of foraminal stenosis or removal of posterolateral soft disc fragments. It maintains the motion in the affected segment and does not cause major instability [69]. Due to the nature of the approach it also has a lower complication rate when compared to anterior procedures [7, 68]. There is a large body of evidence which suggests that it is an effective procedure for cervical monoradiculopathy [70].

Kumar et al. reviewed 89 patients treated with laminoforaminotomy for cervical spondylotic radiculopathy caused by osteophytes [71]. Patients with disc herniation were excluded. Good or excellent results were obtained in 95.5% of patients, a mean followup of 8.6 months using Odom's criteria. Repeat surgery for recurrence was required in 6.7% of cases. Davis reviewed 170 patients who underwent laminoforaminotomy for cervical radiculopathy [72]. Followup, at a mean of 15 years, revealed good or excellent outcomes in 86% of patients, based on Prolo score. There was a 6% recurrence rate with most occurring within the first 3 years of the index surgery. Herkowitz et al. performed a comparison of laminoforaminotomy with ACDF to treat of cervical herniated discs causing radiculopathy in 33 patients [7]. Good and excellent results were reported in 94% of the ACDF group and 75% of the laminoforaminotomy group at a mean followup of 4.2 years. The difference, however, was not statistically significant. 

Shorter duration of the operation and fewer complications, compared to anterior surgery, have been reported as major advantages of posterior laminoforaminotomy [68]. However, complications of this technique include neurological damage, infection, and recurrence of symptoms [71, 72]. A major limitation is that it does not allow removal of offending lesions located medioventral to the nerve root [68].

### 3.2. Anterior Cervical Discectomy

Anterior cervical discectomy and fusion (ACDF) is suggested for the treatment of single-level degenerative cervical radiculopathy for compressive lesions medioventral to the nerve root [5]. Despite providing better access to certain compressive pathologies this technique has the potential for many complications [73]. These relate to the anterior approach itself, graft-related complications, and spinal instability as discussed in the previous section. Despite these complications, many of studies have demonstrated ACDF to be an effective way of alleviating radicular symptoms.

Peolsson et al. reported 34 patients that underwent anterior decompression for cervical radiculopathy with 3-year followup [74]. All patients had an improvement in Visual Analogue Scale, Neck Disability Index scores, and sensory deficit. Korinth et al. reviewed 292 patients with cervical soft disc disease causing radiculopathy at a single level [68]. They compared anterior cervical discectomy using a polymethylacrylate spacer with a posterior laminoforaminotomy procedure. Good and excellent results were found to be statistically different between the anterior (93.6%) and posterior (85.1%) groups in favour of the anterior approach using Odom's criteria at a mean followup of 6.1 years. 

As previously discussed, iliac crest autograft is the gold standard but other fusion techniques may be utilised, each with their own benefits and complications [46]. In the treatment of single-level cervical radiculopathy, ACDF with plate fixation demonstrates similar clinical outcomes and fusion rates to ACDF without plate fixation [75–77]. However, the use of a cervical plate can improve sagittal alignment after ACDF [75–77]. Evidence suggests that plate stabilization may be indicated for some patients undergoing multilevel ACDF for radiculopathy. There is a paucity of evidence linking this practice to significant improvement in clinical outcomes [5].

In recent times, much attention has been focused on the use of cervical disc arthroplasty in an attempt to preserve motion segments. Short-term outcomes suggested comparable efficacy to ACDF for the treatment of single-level degenerative cervical radiculopathy [78, 79]. In the longer-term, Quam et al. reported on 21 patients who underwent 1 or 2 level cervical disc arthroplasty for radiculopathy [80]. At 8 years followup, the Bryan cervical disc arthroplasty maintained favourable clinical and radiological results, with preservation of movement and satisfactory clinical outcome in the majority of cases. However, 48% operated segments developed heterotopic ossification causing restricted range of movement of the prosthesis.

## 4. Conclusion

Cervical spondylotic myelopathy and radiculopathy are common disorders which can lead to significant clinical morbidity. Numerous surgical techniques exist to alleviate symptoms, which are achieved through anterior, posterior, or circumferential approaches. Under most circumstances, one approach will produce optimal results. The surgical plan should be tailored to address each individual's unique clinical circumstance.

When considering surgical outcomes for CSM, it is important to remember that regardless of surgical technique employed, results of operative treatment generally are better in patients who undergo early decompression. In a prospective study of 146 patients with cervical spondylotic myelopathy, Suri et al. noted that patients with less than a one-year duration of symptoms showed significantly greater motor recovery following operation than did those with a longer duration of symptoms [81]. This finding is supported by numerous other studies [82, 83]. Conversely, the symptoms for most patients with degenerative cervical radiculopathy will be self-limited and will resolve spontaneously over a variable length of time without specific treatment [5]. Surgical intervention, however, can lead to rapid relief of symptoms of cervical radiculopathy compared to conservative measures alone [84, 85].

An extensive review of the current peer-reviewed literature does not provide an evidence base to indicate whether anterior or posterior surgery yields superior short- and long-term results for both CSM and cervical radiculopathy. Well-designed prospective randomised-control trials involving patients with these clinical scenarios could help to properly evaluate this.
